# Seed dispersal of wild radishes and its association with within-population spatial distribution

**DOI:** 10.1186/s12898-020-00297-4

**Published:** 2020-05-11

**Authors:** J. Ziffer-Berger, Y. Waitz, E. Behar, O. Ben Joseph, L. Bezalel, H. Wasserstrom, P. K. Bajpai, S. Bhattacharya, F. Przesdzink, E. Westberg, K. Mummenhoff, O. Barazani

**Affiliations:** 1 Department of Biology, Levinsky College of Education, 15 Shoshana Persitz St, 6937808 Tel Aviv, Israel; 2 Herbarium, Steinhardt Museum of Natural History, 12 Klausner St, 6901127 Tel Aviv, Israel; 3 Institute of Plant Sciences, Agricultural Research Organization, 7505101 Rishon LeZion, Israel; 4 The Hebrew University of Jerusalem, Givat Ram, 91904 Jerusalem, Israel; 5 Department of Biology, University of Osnabrück, Osnabrück, Germany; 6 KWS AG, Einbeck, Germany

**Keywords:** Anemochory, Dispersal, Hydrochory, Indehiscent fruits, Long- vs. short-range dispersal

## Abstract

**Background:**

The wild radishes, *Raphanus raphanistrum* and *R. pugioniformis* (Brassicaceae) are native to the East Mediterranean region. However, whereas *R. raphanistrum* is widely distributed worldwide, the endemic *R. pugioniformis* is limited to specific habitats. In *R. raphanistrum* the diaspores of the indehiscent fruits comprise glabrous, light, single-seeded segments, whereas the intact fruits of *R. pugioniformis* are heavy and covered with spiny backward-pointing trichomes. We aimed to investigate whether the structure of the diaspores was directly associated with long- and short-range dispersal in *R. raphanistrum* and *R. pugioniformis*, respectively. We further surveyed within-population spatial distributions, to test the hypothesis that short- and long-range dispersal contribute to a patchy vs. uniform distribution patterns of *R. pugioniformis* and *R. raphanistrum*, respectively.

**Results:**

The results indicated that dispersal by wind and run-off water was substantially lower for diaspores of *R. pugioniformis* than for those of *R. raphanistrum* diaspores. Supporting the hypothesis that backward-pointing trichomes promote adherence to soil particles, the displacement on soil surface of *R. pugioniformis* fruits depended on their orientation relative to wind direction. Furthermore, trichome removal from fruits of *R. pugioniformis* significantly reduced wind velocity needed to remove fruits that were placed on soils typical of the species’ natural habitats. The spatial-distribution survey results indicated a patchy distribution of *R. pugioniformis* populations as compared with the more uniform arrangement in the studied populations of *R. raphanistrum*; consistent with the unidirectional vs. homogeneous wind dispersal of the respective diaspores, with respect to wind direction. In addition, *R. pugioniformis* population sizes changed less between years than those of *R. raphanistrum*.

**Conclusions:**

Overall, our results indicate that fruit structure is strongly linked to dispersal ability and spatial distribution of the two closely related wild radish species. Whereas *R. raphanistrum* inhabits homogenous sandy soil habitats, the distribution range of *R. pugioniformis* includes heterogeneous environments in which growth niches are scarcer. We suggest that the different modes of dispersal have evolved as adaptive traits appropriate to the species’ specific habitats.

## Background

Seed dispersal ability determines the probability that a seed may reach an appropriate environment where the germinating plant can maximize its fitness [1, 2]. The mode of seed dispersal (short- vs. long-range), which is influenced by seed and fruit traits, is generally associated with the scale of plant distribution, population structure and density, and variations in spatial distribution within and between generations [3–7]. Moreover, it was recently suggested that traits that increase seed dispersal ability in the Brassicaceae are associated with high speciation rates [8]. Thus, long-range dispersal, which is assisted by wind, water or animals can significantly influence population dynamics as well as evolutionary processes of lineage diversification [8–10].

In the Brassicaceae, differing dispersal mechanisms characterize dehiscent fruits, from which seeds are freely released to the environment, and indehiscent ones whose dispersal segments contain one or few seeds. In dehiscent fruits, dispersal of seeds can be achieved by ballistic mechanisms, [e.g. 11, 12] whereas in indehiscent fruits it is achieved through morphological attributes of the diaspores. In fruits of *Cakile* for example, traits of the indehiscent dispersal segment promote long-range seed dispersal [13, 14]. However, it seems likely that there is a trade-off between the relatively heavy weight of the indehiscent dispersal units and long-range dispersal [15, 16].

**Fig. 1:**
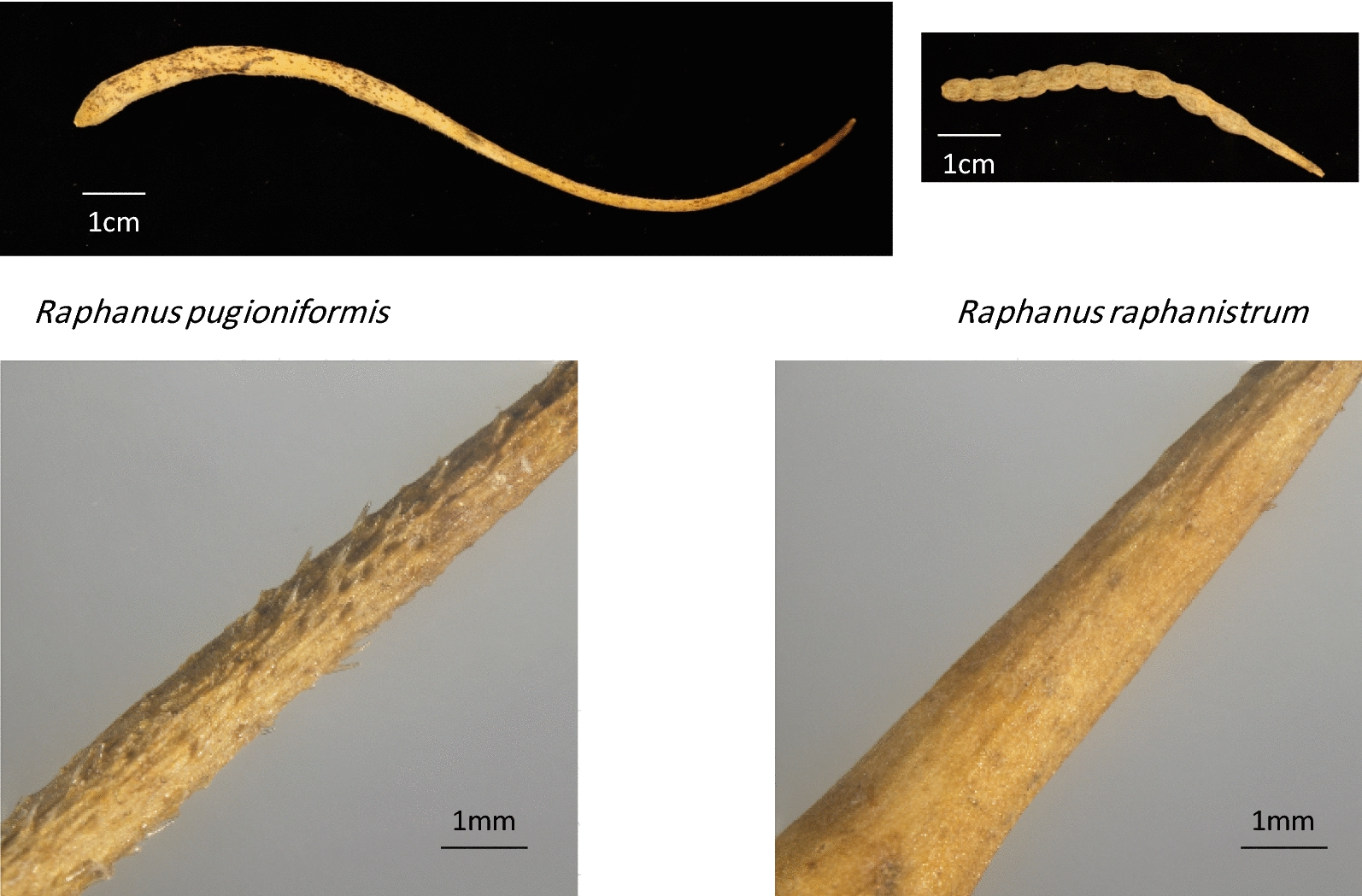
Intact fruits of wild radishes and backward directed (towards distal end) trichomes on the surface of *R. pugioniformis* fruits

**Fig. 2:**
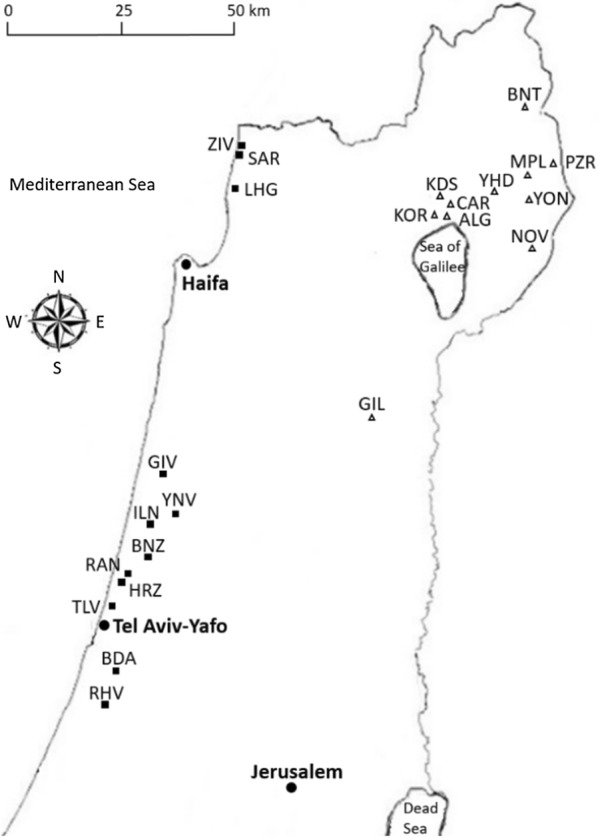
Mapped populations of wild radishes in Israel, showing the distribution ranges of the two allopatric closely related species *R. raphanistrum* (squares) and *R. pugioniformis* (triangles). The names, geographical and environmental characteristics of the various sites are listed in Table 1

In *Raphanus* (tribe Brassiceae), seeds are enclosed in indehiscent lignified fruits (Fig. 1). In the East Mediterranean area, the genus *Raphanus* includes two closely related annual species: *R. raphanistrum* L. and *R. pugioniformis* DC. [17]. The distribution ranges of these two East Mediterranean species do not overlap: *R. raphanistrum* is widely distributed in fragmented habitats along the coastal plain, in relatively homogenous sandy soil; habitats that differ slightly in annual rainfall (Table 1, Fig. 2). Furthermore, *R. raphanistrum* is widely distributed around the Mediterranean Basin, which is its native range, but also as a cosmopolitan weed or as a ruderal species. However, the endemic *R. pugioniformis* is limited to northern Israel and southern Lebanon and Syria, where it grows on either terra rossa (chromic luvisols or rhodustalfs, by the FAO and USDA soil taxonomy, respectively) or basaltic soils. After detachment from the maternal plant during late spring and early summer *R. raphanistrum* fruits usually break into several one-seeded light-weight dispersal units, whereas the relatively long and heavy fruits of *R. pugioniformis*, which, typically contain four to six seeds, abscise completely and remain intact (Fig. 1 and Additional file 1: Fig. S1).

**Table 1:** Sites of the mapped populations of wild radishes: their geographical and environmental characteristics

Site	Coordinates		Altitude (m a.s.l.)	Soil type	Average rainfall (mm year^−1^)
	N	E			
*R. raphanistrum*					
Gesher haZiv (ZIV)	33°02′32.44″	35°06′35.81″	45	Hamra	583
Sa’ar (SAR)	33°01′38.03″	35°06′18.71″	30	Hamra	583
Lohamei haGeta’ot (LHG)	32°57′46.92″	35°05′39.69″	34	Hamra	611
Giva’at Haim (GIV)	32°23′41.13″	34°55′54.15″	45	Hamra	576
Yanuv (YNV)	32°18′44.37″	34°57′16.75″	48	Hamra	653
Ilanot (ILN)	32°17′29.91″	34°53′55.25″	48	Hamra	558
Bnei Zion (BNZ)	32°13′2.89″	34°53′2.601″	42	Hamra	558
Ra’anana (RAN)	32°11′27.80″	34°50′45.73″	57	Hamra	527
Herzelia (HRZ)	32°10′44.36″	34°49′51.82″	27	Hamra	527
Tel Aviv (TLV)	32°07′39.29″	34°48′27.78″	48	Hamra	583
Bet Dagan (BDA)	32° 46′ 39.0′’	35° 39′ 28.0″	50	Hamra	524
Rehovot (RHV)	31°55′7.49″	34°47′36.76″	59	Hamra	572
*R. pugioniformis*					
Bental (BNT)	33°07′21.42″	35°46′47.57″	1010	Basalt	786
Pzura (PZR)	33°00′36.69″	35°50′5.13″	860	Basalt	707
Hamapalim (MPL)	32°59′19.76″	35°46′9.38″	637	Basalt	630
Yehudia (YHD)	32°57′10.01″	35°42′23.02″	254	Basalt	549
Yonathan (YON)	32°56′15.85″	35°47′11.64″	561	Basalt	626
Karei Deshe (KDS)	32°56′38.17″	35°34′4.44″	314	Basalt	578
Carkom (CAR)	32°55′47.17″	35°36′20.29″	124	Basalt	512
Korazim (KOR)	32°54′44.53″	35°33′59.87″	68	Basalt	522
Almagor (ALG)	32°54′24.57″	35°35′47.41″	− 41	Basalt	485
Nov (NOV)	32°50′4.251″	35°47′51.28″	441	Basalt	542
Gilboa (GIL)	32°30′15.95″	35°24′43.52″	436	Terra rossa^a^	385

The locations of the sites are presented on a map in Fig. 2

^a^Terra rossa is termed chromic luvisols or rhodustalfs, by the FAO and USDA soil taxonomy, respectively

We hypothesize that long-range dispersal can contribute for the wide distribution of *R. raphanistrum*, both local and worldwide; this contrasts with the restricted, sub-endemic distribution of *R. pugioniformis*, which can be assisted by its relatively heavy dispersal unit. To test this hypothesis, we conducted experiments on wind dispersal (anemochory) and possible dispersal by run-off water (hydrochory). In addition, we hypothesized that the presence of backward pointing trichomes on the pericarp of *R. pugioniformis* (Fig. 1) increased adherence to the soil surface, as was suggested for *Lachnoloma lehmannii* (Brassicaceae) [18]. As the fruits of *R. pugioniformis* drop of the maternal plant immediately after maturation and remain at the vicinity of the maternal plant (Additional file 1: Fig. S1A) we excluded the possibility of fur-epi-zoochory. Furthermore, field observations showed that rodents consume the seeds by breaking the pericarp (Additional file 1: Fig. S1B), excluding the possibility of endo-zoochory. To evaluate the above two hypotheses, we further surveyed population spatial structure of the two respective species in various sites, in the expectation that short-range dispersal of *R. pugioniformis* would promote a patchy distribution pattern in its native heterogeneous environments, in contrast to the uniformly wide distribution of *R. raphanistrum* (Additional file 1: Fig. S2). Thus, this research study, that associate fruit morphology to dispersal and distribution arrangement, aims at understanding the ecological role of evolutionary processes that lead to different dispersal mechanisms of two closely related species.

## Methods

### Plant material

In a botanical survey in 2015 we mapped 10–12 populations of *R. raphanistrum* and *R. pugioniformis* along their distribution range in Israel (Table 1; Fig. 2). In late spring (May 2015), we collected mature fruits from 25 to 30 maternal plants in each population.

Seeds were recovered from a single fruit of each maternal plant and pooled to form a single seed lot. The seeds were set on moistened filter paper in 9-cm Petri dishes (20 seeds per dish, 60 seeds in total), and germinated in a growth chamber at 25 °C 8/16-h day/night photoperiod. All the seedlings were randomly picked and transferred to Israel’s Agricultural Research Organization’s field site (32° 46′ 39″ N, 35° 39′ 28″ E, ca. 50 m above sea level; ~ 520 mm annual rainfall) in December 2015. Twenty-five plants of each species were grown in separate plots and covered with insect-proof nets to prevent cross-pollination; pollination was achieved by introducing bumble bees (BioBee, Israel) when the plants reached the flowering stage in March 2016. Mature fruits were collected on a single day in early-June 2016, i.e. late spring. In light of the reduced maternal effect on the progeny under identical field conditions, the matured fruits were used for morphological assessment (size and weight of fruits) and dispersal experiments as described below. For this purpose, we pooled 20 fruits of each population into a single lot for each species.

### Dispersal by wind

Dispersal by wind (anemochory) was tested on fruits of each species with a custom-built device that comprised a fan, a wind tuned to homogenize airflow, an anemometer placed above the sample, and a camera to record the experiment. Diaspores of either *R. raphanistrum* or *R. pugioniformis* were placed 30 cm from a fan-based wind source. The wind velocity (m s^−1^) was increased gradually and recorded by the anemometer (Testo 410i; Testo Inc.) until the fruit was detached from the substrate. Three soil types were used as a substrate, representing soils in the natural habitats of the two species (Table 1): loam sand (‘hamra’), terra rossa, and basaltic soil. Wind velocity causing removal (WVR) was tested when fruits were positioned perpendicular and parallel to the fan (Additional file 1: Fig. S3), hypothesizing that the position of trichomes against wind direction can influence the dispersal ability. Thus, because trichomes on fruits of *R. pugioniformis* point towards its distal edge, and because fruits of both species are curved (Fig. 1), WVR was measured when fruits were positioned in parallel (designated as 0 and 180°); and perpendicular position (designated as 90 and 270°) in relation to the fan (Additional file 1: Fig. S3). The same setup was tested with an additional group *R. pugioniformis* fruits from which trichomes had been removed with sandpaper. The WVR was determined 15 times for each orientation (0, 90°, 180° and 270°) and each substrate (hamra, terra rossa, basalt), for 10 intact fruits of each species.

### Dispersal by run-off water

Dispersal by run-off water (hydrochory) was tested generally as described by Garcia-Fayos et al. [19] and with a modified experimental set-up as described by Arshad et al. [20]; the tests were applied to intact fruits and single-seed dispersal segments of *R. raphanistrum*, and to fruits of *R. pugioniformis*. Rainfall and subsequent run-off water flow were simulated by vertically releasing water drops onto a (10 × 20)-cm^2^ impact area of a sloping (11°) ramp covered with 40-grade sandpaper, at a constant rate of 2 L min^−1^, which simulated the natural flow of run-off water from a larger area than the experimental setup. The sandpaper grade was selected to mimic the minimal surface roughness (400 µm) that would prevent rolling of the diaspores in the absence of water flow. Dispersal was indicated and recorded according to shifting of dispersal units from their initial positions. Dispersal by run-off water was tested on 10 fruits at each of three independent experiments, i.e. a total of 30 randomly selected dispersal units of each species.

### Determining the spatial pattern at the natural habitats

In 2017, at each site (Table 1) we divided each of four randomly designated (10 × 10)-m^2^ plots into four quadrants. The coordinates of the centre of each quadrant were determined with a GPS system, to enable a second survey in the following year. The first survey covered 12 populations of *R. raphanistrum* and 11 populations of *R. pugioniformis*; in the following year plants were not found in three sites of *R. raphanistrum* (GIV, ILN and RHV) and at two sites of *R. pugioniformis* (ALG and CAR) (c.f. Table 1 and Fig. 2), so that the second survey covered nine populations of each species. The surveys were conducted in February 2017 and 2018, at the peak of the growing season.

The number of individual plants in each quadrant was determined and summed to determine the total number of plants in each plot. To test the distribution pattern, we applied the Chi-squared test, based on the null hypothesis that the default distribution of plants is uniform, and all quadrants include an equal number of plants. We compared the expected, default uniform distribution with the observed one (designated as *EOr*) by means of Pearson’s Chi-squared test formula: $\mathop \sum \nolimits_{i = 1}^{4} \frac{{\left( {dOi - {\text{d}}Ei} \right)^{2} }}{{dE_{i} }}$, in which *dOi* and *dEi*, respectively, are the observed and expected numbers of individuals. Based on the formula, uniform to patchy distribution patterns are demonstrated in lowest to highest *EOr* values. Year-on-year changes in the populations were assessed by subtracting the observed number of individuals in each plot in the second year from that in the first. In addition, we estimated year-on-year changes in the distribution pattern by subtracting the expected number of individuals of the second year from this of the first (Δ*E*) for each plot separately, indicating on negative or positive growth in population size. The spatial pattern analysis included univariate analysis, in which the relation between the number of individuals in each quadrant and the total number of counted individuals in the (10 × 10)-m^2^ plot was assigned as a weight variable. The data were *log* transformed prior to statistical analysis because of the large span in the order of magnitude in the number of individual plants per plot.

### Data analysis

We used JMP 13.1.0 (SAS Institute Inc.) ANOVA post hoc tests for the statistical analyses. For the dispersal experiment, the species (*R. raphanistrum* and *R. pugioniformis*), substrate (hamra, terra rossa, and basaltic), and presence or absence of trichomes were assigned as independent variables.

## Results

Fruits of *R. pugioniformis* were significantly 2.4 times as heavy (*t*-test: *N *= 25, *F *= 21.118, *P *< 0.0001) and 1.7 times as long (*t*-test: *N *= 25, *F *= 107.741, *P *< 0.0001) as those of *R. raphanistrum*. The respective weights and lengths of *R. pugioniformis* and *R. raphanistrum* were 179.15 ± 19.39 mg and 6.7 ± 0.49 cm, respectively; and 74.75 ± 11.83 mg and 3.9 ± 0.24 cm, respectively.

### Dispersal of fruits by wind

**Fig. 3:**
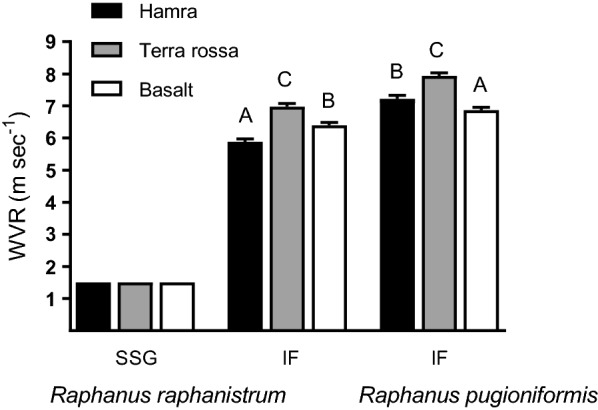
The effect of the substrate on wind velocity causing removal (WVR) of single-seeded segments (SSG) of *R. raphanistrum* and intact fruits (IF) of *R. raphanistrum* and *R. pugioniformis*. Different letters above bars indicate significantly different results for each species separately (Tukey HSD, *P * < 0.05)

Preliminary experiments indicated that single-seeded segments of *R. raphanistrum* detached from the substrate at a wind speed below 1.5 m s^−1^, which was substantially lower than that recorded for removal of intact fruits of *R. raphanistrum* and *R. pugioniformis* (Fig. 3). Therefore, in light of their low threshold of wind-velocity for removal (WVR), single-seeded segments of *R. raphanistrum* were not included in the subsequent wind-dispersal analysis, and we focused on intact fruits.

In general, 1.12 times higher wind velocities were needed to remove fruits of *R. pugioniformis* from the various soil types than those for *R. raphanistrum* (*t*-test: *F *= 66.742; *P *< 0.0001). Results of one-way ANOVA indicated that only fruit weight had a significant effect on the wind velocity needed to remove *R. raphanistrum* fruits (Table 2). For both species the soil substrate had a significant effect on WVR (Table 2): on terra rossa soil, higher wind velocities were needed to remove fruits of both *R. pugioniformis* (7.9 m s^−1^) and *R. raphanistrum* (6.9 m s^−1^) than were needed on basaltic soil (6.9 and 6.4 m s^−1^ for *R. pugioniformis* and *R. raphanistrum*, respectively) (Fig. 3).

**Table 2:** Results of one-way ANOVA of the effects on wind velocity causing removal (WVR), of fruit weight, soil type and fruit orientation (angle)

	*R. raphanistrum*			*R. pugioniformis*		
	*F*	*P*	*R*^2^	*F*	*P*	*R*^2^
Weight	95.143	< 0.0001	0.41	1.554	0.2130	0.26
Soil	31.320	< 0.0001	0.11	28.351	< 0.0001	0.04
Angle	2.808	0.0391	0.02	256.713	< 0.0001	0.36

**Fig. 4:**
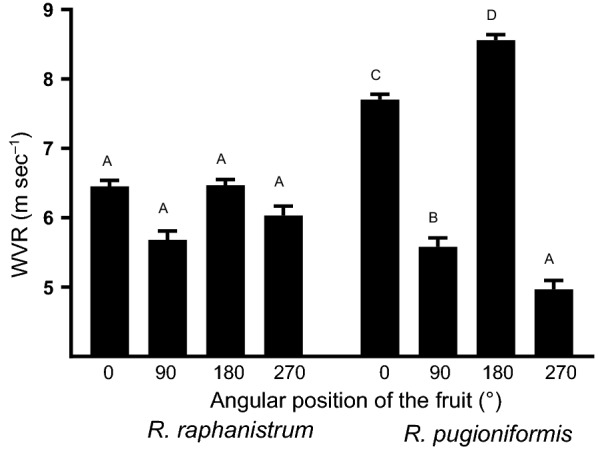
The effect of orientation (cf. Additional file 1: Fig. S3) on wind velocity causing removal (WVR) of fruits of wild radishes. Different letters above bars indicate significantly different results for each species separately (Tukey HSD, *P *< 0.05). Single-seed diaspores of *R. raphanistrum* were not included in this analysis because of their low WVR threshold (see Fig. 3)

**Fig. 5:**
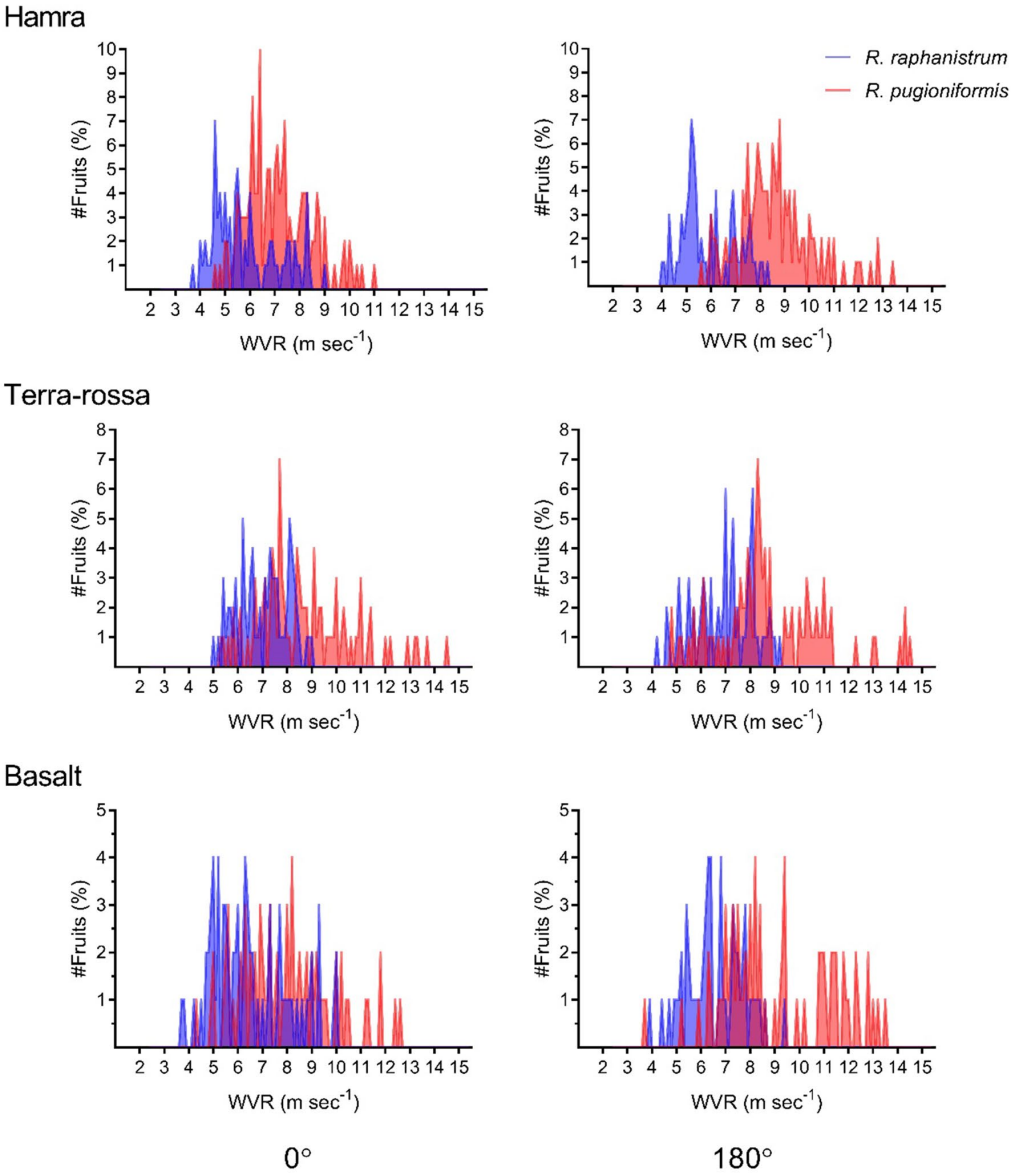
Variation in wind velocity causing removal (WVR) of fruits of *R. raphanistrum* and *R. pugioniformis* on the three soil types. Results represent the percentages of fruits detached at various wind velocities. Single-seed diaspores of *R. raphanistrum* were not included in this analysis because of their low WVR threshold (see Fig. 3)

In all three-soil types, WVR of fruits of *R. pugioniformis* was influenced by the fruit’s parallel orientation relative to the wind direction, unlike that of *R. raphanistrum* fruits (Fig. 4). Frequency plots of the WVR results for the 0 and 180° orientations revealed narrower variation among fruits of *R. raphanistrum* than among those of *R. pugioniformis* (Fig. 5). In general, significantly 1.1 times lower wind velocity was sufficient to remove parallel oriented fruits of *R. pugioniformis* when trichomes faced the wind direction than in the opposite orientation (i.e., orientations of 0 or 180°, respectively; wind speeds of 7.7 and 8.6 m s^−1^, respectively). In light of these results, we further tested the effect of trichome removal and observed significant differences in WVR between fruits with and without trichomes (7.5 ± 0.07 and 6.9 ± 0.10 m s^−1^, respectively; *t*-test, *F *= 23.947; *P *< 0.0001). Further analysis showed that trichome removal significantly reduced WVR when fruits were set at either 0 or 180° orientations on terra rossa, but not on basaltic soil (Tukey HSD test; Additional file 1: Fig. S4).

### Run-off water dispersal

None of the *R. pugioniformis* fruits were displaced by simulated run-off water flowing at 2 L min^−1^, whereas 76.6 ± 7.7% of the single-seeded dispersal segments of *R. raphanistrum* were moved down the slope (Additional file 1: Fig. S5).

### Spatial distribution of wild radishes in their natural habitats

**Fig. 6:**
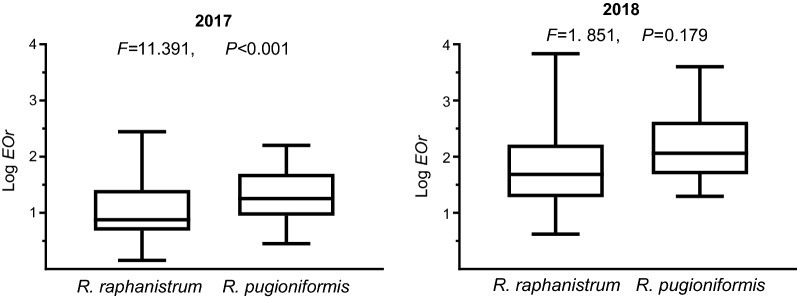
Dispersal patterns of wild radishes in a 2-year survey. Results are presented in a Whisker diagram showing the means with upper and lower variability of *EOr* values; results of statistical analyses comparing the two species are presented above the diagram

The 2017 survey found significant differences between the *EOr* values of the respective species (ANOVA, *F *=11.391, *P *< 0.001), but the 2018 survey did not (*F *=1. 851, *P *= 0.179). In both years *EOr* values of *R. pugioniformis* showed less variation than those of *R. raphanistrum* (Fig. 6), which was indicative of the patchy distribution of the former compared with the more uniform distribution pattern of the latter.

**Fig. 7:**
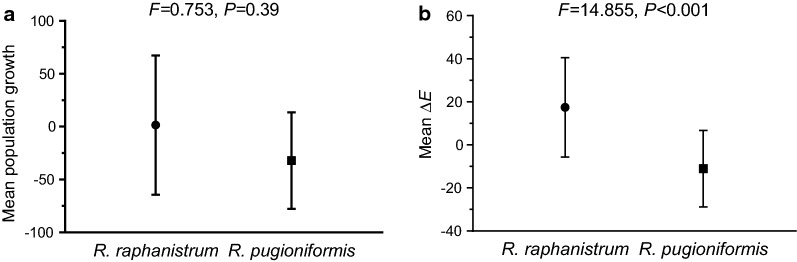
The increase in population size (**a**) and the year-on-year change in the distribution pattern (**b**) of wild radishes. Results are presented as means, with the upper and lower confidence intervals (95%). Results of statistical analyses comparing the two species are presented above the diagrams

Estimates of year-on-year changes in population size revealed no significant differences between species (Fig. 7a). The mean growth of both species was substantially low, at 1.5 and − 32.1 for *R. raphanistrum* and *R. pugioniformis*, respectively, with relatively high variation (Fig. 7a), indicating that on average there was no significant change in population size between the first and second years of the survey. In contrast, there was a significant difference between the species, in their year-on-year changes in distribution patterns, with a mean positive change in Δ*E* value for *R. raphanistrum* (17.5) and a negative change (− 11.0) for *R. pugioniformis* (Fig. 7b).

## Discussion

The results of the two dispersal experiments—by wind and run-off water—showed that there were differences in dispersal ability between the respective single- and multi-seeded indehiscent dispersal units of *R. raphanistrum* and *R. pugioniformis*. In general, the displacement of *R. pugioniformis* fruits by wind or run-off water was substantially lower than those of intact fruits or single-seeded diaspores of *R. raphanistrum* (Table 2, Figs. 3, 4, 5, Additional file 1: Fig. S5). Moreover, the dispersal ability of fruits of *R. raphanistrum* was demonstrated in the wind dispersal experiment, which showed that the wind velocity causing removal (WVR) was not influenced by the orientation of the fruit relative to the fan (Figs. 4 and 5). Characteristics of members of the Brassicaceae provide evidence that the evolution of indehiscence was associated with a tradeoff against long-range dispersal [8]. However, the development of breakable single-seeded dispersal segments in *R. raphanistrum* clearly enhanced long-distance dispersal ability, which can contribute to the wide distribution of this species in the East Mediterranean region, as well as its invasive capabilities outside its natural distribution range.

In the present study displacement of *R. pugioniformis* fruits was influenced by fruit orientation and soil type, but not by fruit weight (Table 2; Figs. 4, 5 and Additional file 1: Fig. S4). Moreover, stronger wind was needed to move *R. pugioniformis* fruits placed on terra rossa soil than those on basaltic soil (Fig. 3). Both terra rossa and basaltic soils occur in the natural growing habitats of *R. pugioniformis* (Table 1), but terra rossa contains higher contents of fine-grained clay and silt, whereas the tuffic particles of basaltic soil are substantially bigger [21]. On basaltic soil, WVR was influenced by the orientation of *R. pugioniformis* fruits, rather than that of their trichomes (Additional file 1: Fig. S4). In contrast, trichome removal significantly reduced WVR of fruits set on terra rossa in either orientation (Additional file 1: Fig. S4), which supports the role of trichomes in adherence of the fruits to soil particles [18]. Thus, our overall results suggest that short-range dispersal is a habitat-specific adaptive feature of *R. pugioniformis* fruits.

It has been suggested that adaptation to unpredictable harsh environments in which localized disturbances are rare is more likely to favour short-range dispersal, whereas long-range distribution is expected in more favourable conditions with frequent localized disturbances [22]. Thus, in the heterogeneous habitats of *R. pugioniformis* (Additional file 1: Fig. S2), short-range dispersal can provide an ecological advantage at sites where growth niches among rocks and boulders are relatively scarce. This interpretation is supported by the present survey finding of indications of a patchy spatial distribution within populations of *R. pugioniformis* (Fig. 6), with smaller year-on-year changes in the distribution pattern (Fig. 7). However, it also could be that the patchy distribution resulted from heterogeneity of the habitats rather than from the dispersal ability. Suzuki et al. [3] showed that the patchy distribution of populations of the biennial *Lysimachia rubida* (Primulaceae) in the Bonin Islands was not associated with heterogeneity of the habitats but with seed dispersal and other life-history traits. Thus, short-range dispersal of *R. pugioniformis*, which ensures survival success of an offspring at the site of its maternal plant, could contribute for the spatial patchiness of plant populations.

The results of the present dispersal experiments (Table 2; Figs. 3, 4, 5) suggest that long-range dispersal of single-seeded segments of *R. raphanistrum* through relatively homogenous environments (Table 1) might contribute for the distribution pattern of the species in the East Mediterranean region (Fig. 2 and Additional file 1: Fig. S2). In addition, compared with those of *R. pugioniformis*, populations of *R. raphanistrum* showed a more uniform distribution (Fig. 6), suggesting that dispersal ability also may reduce the likelihood of competition among siblings [5]. This could be highly important in the sandy soils of the East Mediterranean coastal plains (Table 1), with their low organic matter contents and low water-holding capacity [21]. In this context it is important to note that our survey found that populations of *R. raphanistrum* became slightly patchier with passing time (Fig. 7b), which suggests that the spatial distribution could be influenced by disturbance factors. Indeed, populations that were not surveyed in the second year of the present study had been lost because of human activity.

Among the Brassicaceae, indehiscent fruits evolved many times [23], to protect the seeds against harsh arid environments [15]. Specifically such fruits may have evolved for several reasons, including: escape in time and space [24, 25]; protection of seeds from predation [18, 26]; and avoidance of high soil-surface temperatures [18, 27]. Alternatively, fruits may control germination timing via pericarp-imposed dormancy, thereby increasing the likelihood that seedlings be established under favourable conditions [18, 25, 26, 28, 29]. A study of six crucifer species, native to the cold desert environment of north-west China, demonstrated the adaptive value of indehiscence in restricting seed germination [30]. It was suggested that the hard pericarp of *R. raphanistrum* restricts the process of imbibition and thereby affects germination [31]. Thus, in order to understand the ecological consequences of dispersal ability (e.g., reduced competition among siblings, invasive capability), studies of dispersal over short and long distances, as exemplified in the multi- and single-seeded dispersal units of *R. pugioniformis* and *R. raphanistrum*, respectively, should be coupled with more elaborate investigations. Molecular analysis that is in progress will analyse the intra- and inter-population genetic variations that should reflect the differing dispersal strategies and spatial distributions of the two *Raphanus* species. Furthermore, we will specifically analyse the potential role of the pericarp in dormancy [29] and protection from high temperatures.

## Conclusion

Overall, the results of the present study indicate that dispersal ability over long or short distances contribute for the mode of distribution and the within-population uniform or patchy spatial distributions of *R. raphanistrum* and *R. pugioniformis*, respectively. Furthermore, the evolution of differing fruit structures and dispersal strategies in the two closely related species can be directly linked to their adaptive value in the distinct habitats of the two respective species.

## Supplementary information

**Additional file 1.** Additional figures.

## Data Availability

All relevant data supporting our findings are presented in the article and supplementary information.

## Abbreviations

*EOr*: The ratio between the expected (uniform) and observed plant distribution patterns
IF: Intact whole fruit
SSG: Single-seeded fruit segments
WVR: Wind velocity that causes removal

## References

[1] de Casas RR, Willis CG, Donohue K, Colbert J, Baguette M, Benton TG, Bullock JM (2012). Plant dispersal phenotypes: a seed perspective of maternal habitat selection. Dispersal ecology and evolution.

[2] Willson MF, Traveset A, Fenner M (2000). The ecology of seed dispersal. Seeds: the ecology of regeneration in plant communities.

[3] Suzuki RO, Suzuki JI, Kachi N (2005). Change in spatial distribution patterns of a biennial plant between growth stages and generations in a patchy habitat. Ann Bot.

[4] Aguiar MR, Sala OE (1997). Seed distribution constrains the dynamics of the Patagonian steppe. Ecology.

[5] Donohue K (1997). Seed dispersal in *Cakile edentula* var. *lacustris*: decoupling the fitness effects of density and distance from the home site. Oecologia.

[6] Hiebeler D (2000). Populations on fragmented landscapes with spatially structured heterogeneities: landscape generation and local dispersal. Ecology.

[7] Levin SA, Muller-Landau HC, Nathan R, Chave J (2003). The ecology and evolution of seed dispersal: a theoretical perspective. Annu Rev Ecol Evol Syst.

[8] Willis CG, Hall JC, Rubio de Casas R, Wang TY, Donohue K (2014). Diversification and the evolution of dispersal ability in the tribe Brassiceae (Brassicaceae). Ann Bot.

[9] Cain ML, Milligan BG, Strand AE (2000). Long-distance seed dispersal in plant populations. Am J Bot.

[10] Bohrer G, Nathan R, Volis S (2005). Effects of long-distance dispersal for metapopulation survival and genetic structure at ecological time and spatial scales. J Ecol.

[11] Thiede DA, Augspurger CK (1996). Intraspecific variation in seed dispersion of *Lepidium campestre* (Brassicaceae). Am J Bot.

[12] Hayashi M, Gerry SP, Ellerby DJ (2010). The seed dispersal catapult of *Cardamine parviflora* (Brassicaceae) is efficient but unreliable. Am J Bot.

[13] Maun M, Payne A (1989). Fruit and seed polymorphism and its relation to seedling growth in the genus *Cakile*. Can J Bot.

[14] Payne AM, Maun M (1981). Dispersal and floating ability of dimorphic fruit segments of *Cakile edentula* var. *lacustris*. Can J Bot.

[15] Ellner S, Shmida A (1981). Why are adaptations for long-range seed dispersal rare in desert plants?. Oecologia.

[16] Lu JJ, Tan DY, Baskin JM, Baskin CC (2010). Fruit and seed heteromorphism in the cold desert annual ephemeral *Diptychocarpus strictus* (Brassicaceae) and possible adaptive significance. Ann Bot.

[17] Ziffer-Berger J, Hanin N, Fogel T, Mummenhoff K, Barazani O (2015). Molecular phylogeny indicates polyphyly in *Raphanus* L. (Brassicaceae). Edinb J Bot.

[18] Mamut J, Tan DY, Baskin CC, Baskin JM (2014). Role of trichomes and pericarp in the seed biology of the desert annual *Lachnoloma lehmannii* (Brassicaceae). Ecol Res.

[19] Garcia-Fayos P, Bochet E, Cerda A (2010). Seed removal susceptibility through soil erosion shapes vegetation composition. Plant Soil.

[20] Arshad W, Sperber K, Steinbrecher T, Nichols B, Jansen VAA, Leubner-Metzger G, Mummenhoff K (2019). Dispersal biophysics and adaptive significance of dimorphic diaspores in the annual *Aethionema arabicum* (Brassicaceae). New Phytol.

[21] Singer A (2007). The soils of Israel.

[22] Levin SA, Cohen D, Hastings A (1984). Dispersal strategies in patchy environments. Theor Popul Biol.

[23] Muhlhausen A, Lenser T, Mummenhoff K, Theissen G (2013). Evidence that an evolutionary transition from dehiscent to indehiscent fruits in *Lepidium* (Brassicaceae) was caused by a change in the control of valve margin identity genes. Plant J.

[24] Eriksson O (2008). Evolution of seed size and biotic seed dispersal in angiosperms: paleoecological and neoecological evidence. Int J Plant Sci.

[25] Hu X, Yu J, Yang L, Wu Y, Wang Y: Pericarp imposed seed dormancy in *Zygophyllum xanthoxylum* (Bunge) Maxim. favours its adaptation to desert environments. In: Proceedings of the 7th International Herbage Seed Conference: 2010. IHSG. p. 99–103.

[26] Ohadi S, Mashhadi HR, Tavakol-Afshari R (2011). Effects of storage and burial on germination responses of encapsulated and naked seeds of turnipweed *(Rapistrum rugosum*) to light. Weed Sci.

[27] Moreira B, Pausas JG (2012). Tanned or burned: the role of fire in shaping physical seed dormancy. PLoS ONE.

[28] Sperber K, Steinbrecher T, Graeber K, Scherer G, Clausing S, Wiegand N, Hourston JE, Kurre R, Leubner-Metzger G, Mummenhoff K (2017). Fruit fracture biomechanics and the release of *Lepidium didymum* pericarp-imposed mechanical dormancy by fungi. Nat Commun.

[29] Mohammed S, Turečková V, Tarkowská D, Strnad M, Mummenhoff K, Leubner-Metzger G (2019). Pericarp-mediated chemical dormancy controls the fruit germination of the invasive hoary cress (*Lepidium draba*), but not of hairy whitetop (*Lepidium appelianum*). Weed Sci.

[30] Lu JJ, Zhou YM, Tan DY, Baskin CC, Baskin JM (2015). Seed dormancy in six cold desert Brassicaceae species with indehiscent fruits. Seed Sci Res.

[31] Cousens RD, Young KR, Tadayyon A (2010). The role of the persistent fruit wall in seed water regulation in *Raphanus raphanistrum* (Brassicaceae). Ann Bot.

