# Supplementary material for: Seed dispersal of wild radishes and its association with within-population spatial distribution
Source: BMC Ecol. 2020 May 11;20:30. doi: 10.1186/s12898-020-00297-4 (PMC7212605; doi:10.1186/s12898-020-00297-4)
Supplement: Supplementary file 1 — Additional file 1. Additional figures. [file 12898_2020_297_MOESM1_ESM.docx]

**Additional Material**

*Figure S1*. (A) Cluster of one year old fruits of *R. pugioniformis* (marked with yellow arrows), suggesting the assumption of short distance dispersal of the relatively heavy fruits, which fall off the maternal plant; (B) Predation of seeds of *R. raphanistrum* showing that rodents break the pericarp (blue arrows) and consume the seeds without the testa (green arrows). The photos are based on personal observations.

*Figure S2*. Representative populations of *R. raphanistrum* (A-B) and *R. pugioniformis* (C-D) illustrating the heterogeneous habitats of *R. pugioniformis* compared with those of *R. raphanistrum*, and comparing the patchy and homogeneous spatial distributions of the respective species.

*Figure S3*. Fruits were placed on the soil surface in four orientations relative to the fan, as illustrated for fruits of *R. pugioniformis*; the directions of trichomes on the fruit surfaces are illustrated with brown lines.

*Figure S*4. The effect of trichome removal on wind velocity causing removal (WVR) of fruits of *R. pugioniformis* set at two orientations (0 and 180°) relative to the wind direction (cf. Fig. S3). Blue and red bars on the left-hand and central graphs indicate the percentages of fruits with (blue) and without trichomes (red) detached at various wind velocities. Average values of WVR ± standard errors, with results of Tukey HSD test (*P* < 0.05) are presented on the right.

*Figure S5.* Dispersal by run-off water of single-seeded fruit segments of *R. raphanistrum* and intact fruits of *R. raphanistrum* and *R. pugioniformis*. Results represent the percentages (average ± standard error) of dispersal units that were displaced from their initial position by run-off water flowing at 2 L min^-1^ over an 11° slope.
